# A pharmacovigilance study of the association between proton pump inhibitors and tumor adverse events based on the FDA adverse event reporting system database

**DOI:** 10.3389/fphar.2024.1524903

**Published:** 2024-12-19

**Authors:** Ya-Jun Zhang, Dan-Dan Duan, Qian-Yu Tian, Cai-E. Wang, Shu-Xun Wei

**Affiliations:** ^1^ Department of Pharmacy, The First Affiliated Hospital, and College of Clinical Medicine of Henan University of Science and Technology, Luoyang, China; ^2^ Department of Pharmacy, Henan Provincial Corps Hospital of Chinese People’s Armed Police Force, Zhengzhou, China; ^3^ Department of Vascular Surgery, Eastern Hepatobiliary Surgery Hospital, Naval Medical University/Second Military Medical University, Shanghai, China

**Keywords:** proton pump inhibitors, tumor adverse events, FAERS, disproportionality analysis, pharmacovigilance

## Abstract

**Background:**

Proton pump inhibitors (PPIs) are effective treatments for acid-related disorders but may pose tumor risks with long-term use. Current research on PPI-associated tumor adverse events (TAEs) is limited and inconclusive. This study aims to comprehensively analyze the relationship between PPIs and TAEs.

**Methods:**

We analyzed PPI adverse reaction reports from the FDA Adverse Event Reporting System (FAERS) database spanning from 2004 to 2024, focusing on five commonly used PPIs: esomeprazole, pantoprazole, lansoprazole, omeprazole, and rabeprazole. We conducted a disproportionality analysis utilizing the Reporting Odds Ratio (ROR) to identify potential TAEs associated with PPIs. We conducted univariate logistic regression analysis to explore the influencing factors.

**Results:**

A total of 3,133 TAEs were identified, representing 2.36% of all PPI-related adverse events (AEs). The most common TAEs were gastric cancer (19.05%) and malignant neoplasm (7.23%). Disproportionality analysis revealed ten significant TAEs associated with PPIs, including gastric adenocarcinoma and renal cell carcinoma. The median age of those reporting TAEs was 59 (interquartile range [IQR]: 51–70), and 29.70% of them resulted in a fatality. TAEs associated with PPIs were less likely to occur in elderly patients (65–75: OR = 0.91 [0.87–0.95], *p* < 0.001; >75: OR = 0.93 [0.89–0.98], *p* < 0.01).

**Conclusion:**

TAEs constitute a small but significant fraction of PPI-related AEs. This study highlights the need for cautious long-term use of PPIs and further research to understand the underlying mechanisms and risk factors. Clinicians should be aware of the potential tumor risks associated with prolonged PPI treatment.

## Introduction

Proton pump inhibitors (PPIs) are widely prescribed medications for managing acid-related disorders and gastric issues. Over the past few decades, they have demonstrated significant efficacy in reducing stomach acid production. These acid-suppressing agents are well-established treatments for various conditions, including peptic ulcer disease, *H. pylori* (*H. pylori*) eradication, stress ulcer prophylaxis, NSAIDs-associated ulcer disease, Barrett’s esophagus, upper gastrointestinal bleeding, esophagitis, Zollinger-Ellison syndrome, and dyspepsia (Malfertheiner et al., 2017; Nehra et al., 2018). Consequently, PPIs such as omeprazole, esomeprazole, lansoprazole, pantoprazole, and rabeprazole are considered safe and primary treatment options. They function by irreversibly inhibiting the gastric acid pump H+/K + ATPase in the cytoplasmic membranes of parietal cells, leading to a significant reduction in acid secretion into the stomach (Shin and Kim, 2013; Strand et al., 2017).

However, prolonged and profound acid inhibition by PPIs may significantly elevate systemic gastrin levels, resulting in hypergastrinemia (Lundell et al., 2015). This condition may also contribute to an increased risk of gastric cancer (Fossmark et al., 2019; Cavalcoli et al., 2015; Nandy et al., 2016; Perry et al., 2020; Jianu et al., 2012). While some studies have described tumor adverse events (TAEs) associated with long-term PPI use, the existing evidence is still insufficient, underscoring the urgent need for further research into the relationship between PPIs and TAEs (Segna et al., 2021; Guo et al., 2023; Kamal et al., 2021). Thus, it is crucial to focus on the potential serious adverse effects of long-term PPI use, particularly those associated with tumor risks, beyond more common minor side effects such as diarrhea, nausea, vomiting, fatigue, constipation, and headaches (Nehra et al., 2018).

To comprehensively analyze the relationship and influencing factors between PPIs and TAEs, we conducted a study utilizing PPI reports from the FDA Adverse Event Reporting System (FAERS) database spanning from 2004 to 2024. The FAERS is a large database comprising international, spontaneous, and voluntary reports of adverse reactions associated with drugs, natural substances, vaccines, and medical devices approved by the FDA. The adverse reactions included in this database provide substantial evidence for reviewing side effects, particularly the TAEs associated with PPIs highlighted in this study. We collected data on TAEs, performed disproportionality analyses to identify these events, and explored potential influencing factors associated with TAEs that are highly correlated with PPIs. This study aims to provide a comprehensive understanding of TAEs associated with PPIs, offering valuable references for clinical practice.

## Methods

### Data source

The FAERS database, a comprehensive pharmacovigilance resource, derives its data from real-world settings. It has been instrumental in studying adverse events (AEs), including those linked to PPIs. By leveraging this extensive database, we can obtain crucial information such as patient demographics, medical history, the timing of AEs, and their outcomes. AEs are encoded using the Preferred Terms (PT) from the Medical Dictionary for Regulatory Activities (MedDRA), which organizes terms into five hierarchical levels. PTs specify medical concepts like symptoms and disease diagnoses. In addition, “High-Level Terms” (HLTs) and “High-Level Group Terms” (HLGTs) are part of the hierarchy. These terms are further categorized into “System Organ Classes” (SOCs) according to elements including purpose, manifestation site, and causation.

### Data processing procedure

**FIGURE 1:**
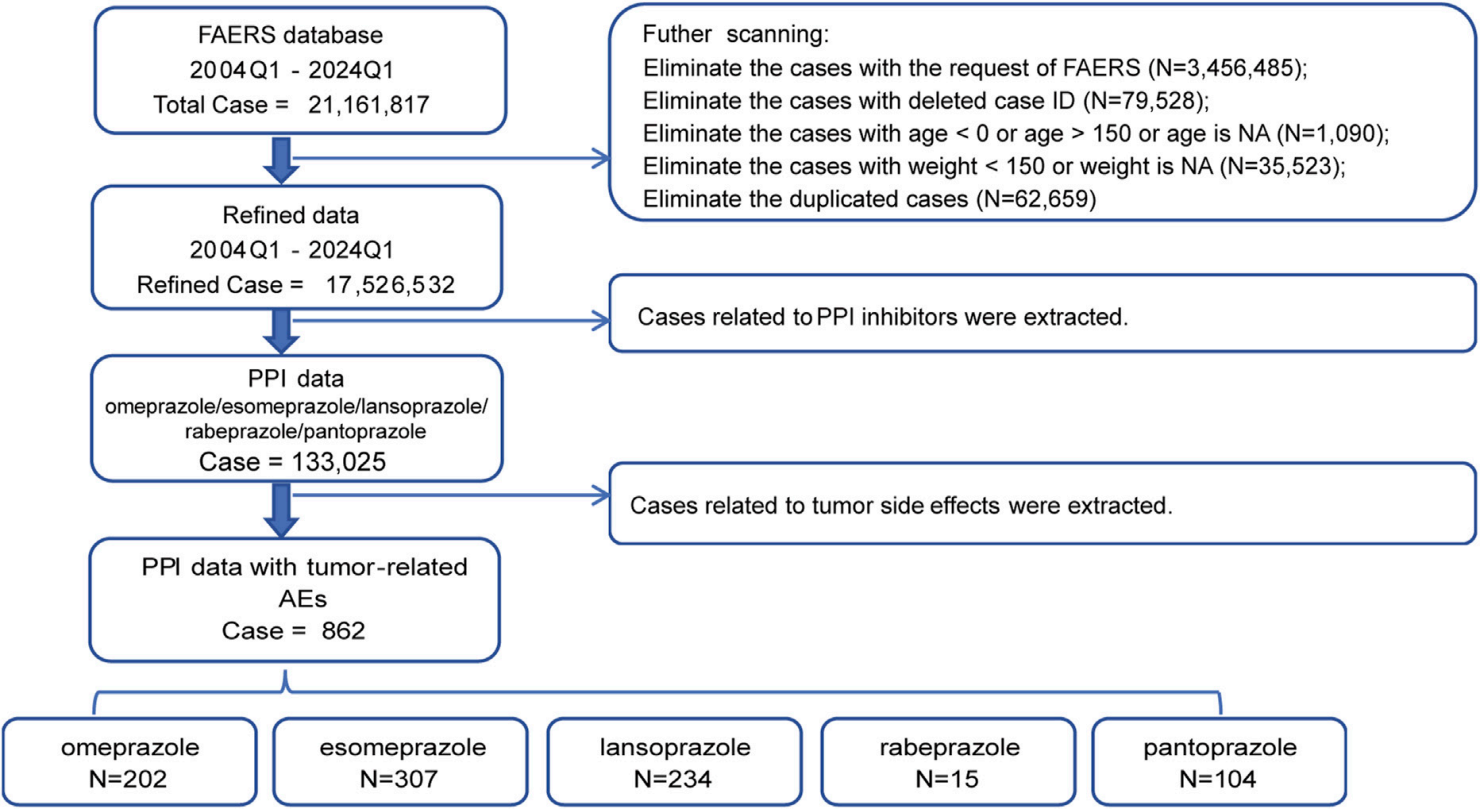
Flow chart showing the selection process of the study for tumor adverse events (TAEs) induced by proton pump inhibitors (PPIs) in the FDA Adverse Event Reporting System (FEARS) analysis.

We concentrated on five PPIs—esomeprazole, pantoprazole, lansoprazole, omeprazole, and rabeprazole—to gather relevant reports for the period of 1 January 2004, to 31 March 2024 from the FAERS Dashboard. Extracted variables included CASEID, age, sex, event date, drug name, and outcome. Reports that did not meet our criteria were excluded: 1) Duplicate records were removed in accordance with FDA guidelines, prioritizing the latest FDA_DT and, in case of ties, the higher PRIMARYID; 2) Samples from FAERS delete files were discarded; 3) Records with ages below 0 or above 150 were excluded; 4) Samples with weights over 150 kg were excluded; 5) All duplicate samples were removed. Figure 1 depicts the comprehensive workflow for data processing.

#### Signal mining

Disproportionality analysis was used to identify potential signals (Caster et al., 2020). In comparison to all other medications in the FAERS database, the Reporting Odds Ratio (ROR) calculates the probability that an adverse event will be reported for a particular drug (Rothman et al., 2004; Sakaeda et al., 2011). To detect signals of tumor adverse reactions in PPI reports, we calculated the ROR and its 95% Confidence Interval (CI):

$$\text{ROR}=\frac{a/c}{b/d}$$

$$95\%\text{ CI}=e^{\ln\left(ROR\right)\pm 1.96\sqrt{\frac{1}{a}+\frac{1\;}{b}+\frac{1}{c}+\frac{1}{d}}.}$$

If at least three TAEs were recorded and the lower bound of the 95% CI of the ROR was more than one, the signal was deemed legitimate and significantly linked with PPI usage (Yan et al., 2022).

### Statistical analysis

The Kaplan-Meier method estimated the event-free probability for PPI-related TAEs. We used the Mann-Whitney *U* test and the Log-rank test to examine the median onset times among the groups. For categorical variables and disproportionality analysis, we employed the Fisher’s exact test or Chi-square test. Potential exposure variables for PPI-related TAEs were investigated, including age, gender, treatment approach, and the severity of results. To determine the odds ratios (OR) for different exposure levels, a univariate logistic regression analysis was carried out. All tests were two-sided, and a P-value <0.05 indicated statistical significance. Statistical analyses and visualizations were performed using R software (version 4.4.1).

## Results

### Tumor adverse events in PPI users: FDA adverse events reporting system (2004–2024)

Using data from the FAERS database spanning the first quarter of 2004 to the first quarter of 2024, we first investigated the incidence of TAEs among patients receiving PPI treatment. After excluding cases where TAEs might have been caused by concomitant medications or other concurrent treatments, we obtained statistical data on TAEs from cases treated with PPIs over nearly 20 years.

**FIGURE 2:**
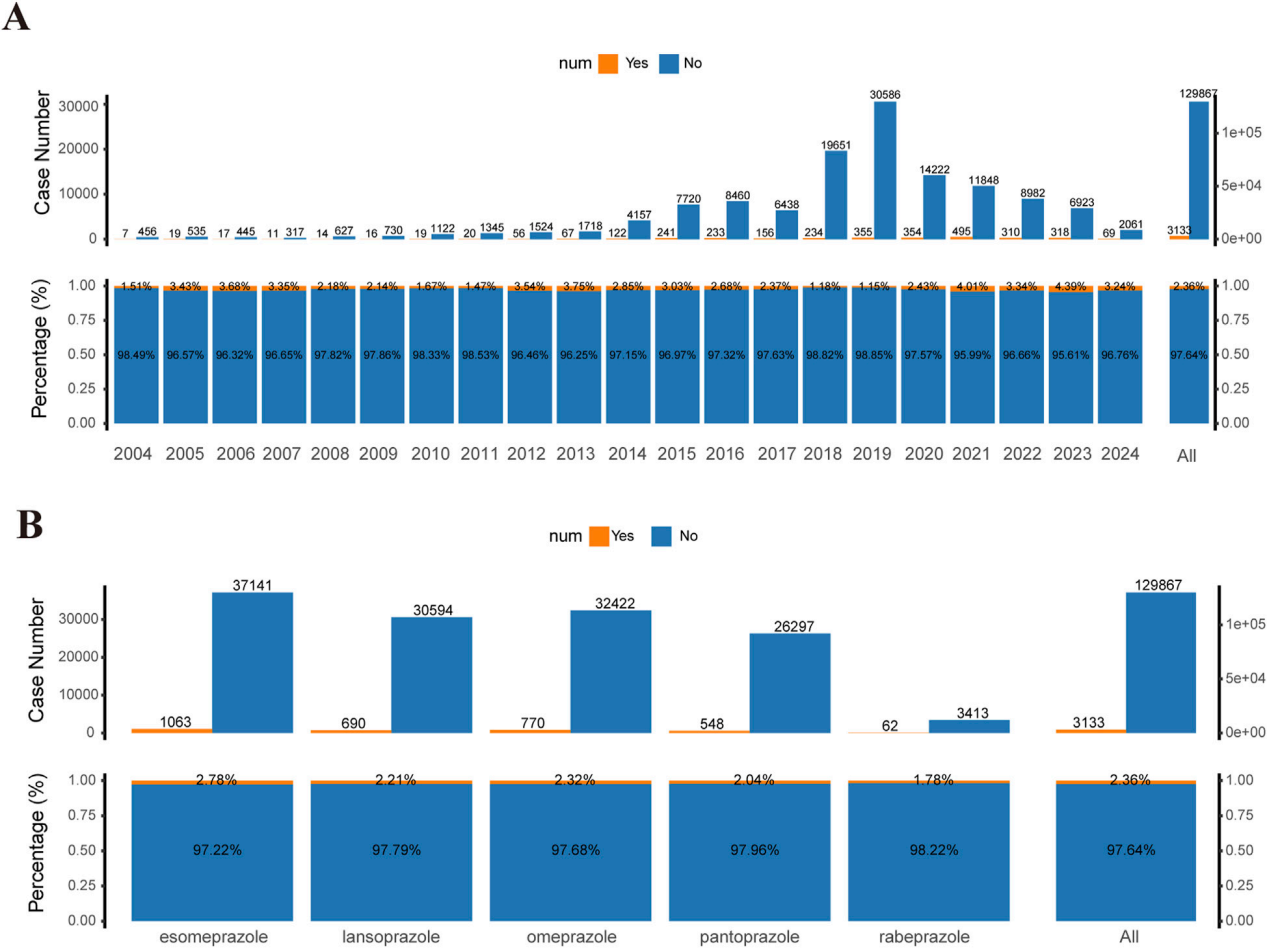
Statistical data of TAEs reported in the FAERS database (2004–2024). **(A)** Top bar chart: Number of tumor-related and non-tumor-related adverse events (AEs) reported for PPIs in the FAERS database from 2004 to 2024 (first quarter). Bottom proportion bar chart: Proportions of tumor-related versus non-tumor-related AEs for the same period. **(B)** Top bar chart: Number of tumor-related and non-tumor-related AEs for different PPI treatment strategies in the FAERS database from 2004 to 2024. Bottom proportion bar chart: Proportions of tumor-related versus non-tumor-related AEs for different PPI treatment groups. PPIs include esomeprazole, lansoprazole, omeprazole, pantoprazole, and rabeprazole.

In all reports of AEs associated with PPIs, the incidence of TAEs only made up a minor portion of the total AEs, with 3,133 cases representing 2.36% of the total cases (3,133/129,867). Moreover, the annual number of TAEs constituted a small portion of the total cases for each year, ranging from 1.15% to 4.39% from 2004 to 2024 (Figure 2A). Additionally, there were differences in the incidence rates of TAEs associated with different PPIs. The proportion of TAEs associated with rabeprazole was 1.78%, while the proportion for esomeprazole was 2.78% (Figure 2B). Overall, these data indicate that, among PPI-associated AEs, TAEs represent a non-negligible component.

### Scanning for TAEs in PPIs

We have compiled the classification of TAEs linked to PPIs use together with the total number of cases reported in the relevant literature (Supplementary Table S1). The top five TAEs with the highest incidence were gastric cancer (N = 582, 19.05%), malignant neoplasm (N = 221, 7.23%), renal cancer (N = 184, 6.02%), gastric adenocarcinoma (N = 142, 4.65%), and breast cancer (N = 132, 4.32%).

**FIGURE 3:**
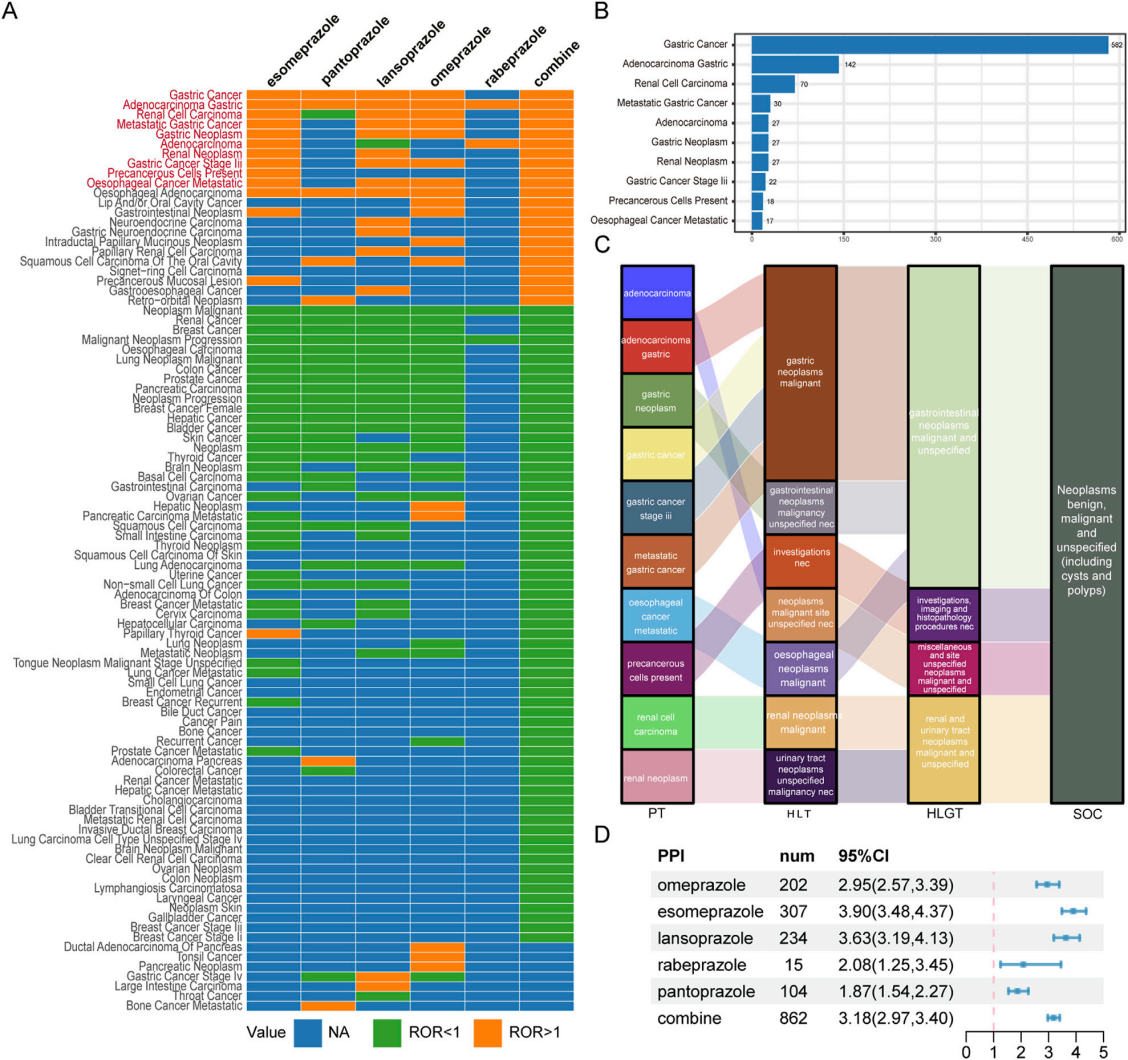
Scanning TAEs associated with PPIs based on the FAERS database. **(A)** This heatmap shows the reporting odds ratio (ROR) of TAEs under different PPI treatments in the FAERS database. Orange indicates ROR values greater than 1, green indicates ROR values less than 1, and blue indicates ROR values that could not be calculated. TAEs labelled with red color meet the criteria that in the combine group, the lower limit of the 95% confidence interval for the ROR greater than 1, and at least 3 cases. **(B)** The bar chart displays the number of reported cases of 10 TAEs under different PPI treatments. **(C)** The Sankey diagram depicts the hierarchical relationship of Preferred Terms (PTs) for 10 categories of TAEs caused by PPIs according to MedDRA. PT stands for Preferred Term, HLT stands for High-Level Term, HLGT stands for High-Level Group Term, and SOC stands for System Organ Class. **(D)** The forest plot shows the ROR of TAEs under different PPI treatments.

Using the comprehensive FAERS database for comparison, we calculated the ROR of TAEs with at least three reported cases for a disproportionality analysis. After applying our effective signal criteria, we found that different TAEs were associated with different PPIs (Figure 3A). Ultimately, in the overall context, ten types of tumor-related PTs that are highly associated with PPI treatment were defined as PPI-associated TAEs: gastric cancer, gastric adenocarcinoma, renal cell carcinoma, metastatic gastric cancer, gastric neoplasm, adenocarcinoma, renal neoplasm, gastric cancer stage III, precancerous cells present, and oesophageal cancer metastatic (Figure 3B). Among these, gastric adenocarcinoma had the highest signal with 142 cases (ROR = 30.27 [25.20, 36.35]), and gastric cancer has the highest number of cases (N = 582). Additionally, there were 70 cases of renal cell carcinoma and 27 cases of renal neoplasm related to the kidney (Supplementary Table S2).

Figure 3C illustrates the affiliation of PPI-associated tumor adverse event PTs with other levels of MedDRA, with the main SOC being neoplasms benign, malignant, and unspecified (including cysts and polyps). Based on the complete FAERS database, we recalculated the ROR for TAEs associated with PPIs. Overall, PPI treatment was substantially linked to the incidence of TAEs (ROR = 3.18 [2.97, 3.40]), although there were differences among the various PPIs (Figure 3D).

### Descriptive analysis of TAEs in PPIs

After screening reports related to PPIs in the FAERS database, we identified 862 cases of TAEs associated with PPIs and conducted a statistical analysis of these cases’ clinical features (Table 1). The median age of those reporting TAEs was 59 (interquartile range [IQR]: 51–70), with a majority of reports concerning males (N = 318, 37%). Most reports originated from the United States (N = 703, 82%).

**TABLE 1:** Characteristics of reports with PPIs-related tumor adverse events (TAEs) from the FAERS database (January 2004-March 2024).

Fatal				
Characteristic	Overall, N = 862^a^	Yes, N = 256^a^	No, N = 606^a^	P value^b^
**Age**	59 (51, 70)	60 (50, 71)	59 (51, 69)	0.4
**Sex**				0.5
female	267 (31%)	86 (34%)	181 (30%)	
male	318 (37%)	93 (36%)	225 (37%)	
missing	277 (32%)	77 (30%)	200 (33%)	
**Reporter Country**				<0.001
GB	19 (2.2%)	12 (4.7%)	7 (1.2%)	
JP	60 (7.0%)	7 (2.7%)	53 (8.7%)	
US	703 (82%)	223 (87%)	480 (79%)	
OTHER	73 (8.5%)	13 (5.1%)	60 (9.9%)	
missing	7 (0.8%)	1 (0.4%)	6 (1.0%)	
**Group**				0.2
esomeprazole	307 (36%)	99 (39%)	208 (34%)	
lansoprazole	234 (27%)	70 (27%)	164 (27%)	
omeprazole	202 (23%)	55 (21%)	147 (24%)	
pantoprazole	104 (12%)	31 (12%)	73 (12%)	
rabeprazole	15 (1.7%)	1 (0.4%)	14 (2.3%)	
**Time to Event**	2,556 (1,070, 4,709)	2,556 (797, 4,908)	2,556 (1,096, 4,547)	0.8
**Year**				0.016
2000–2005	4 (0.5%)	1 (0.4%)	3 (0.5%)	
2005–2010	12 (1.4%)	2 (0.8%)	10 (1.7%)	
2010–2015	19 (2.2%)	3 (1.2%)	16 (2.6%)	
2015–2020	186 (22%)	40 (16%)	146 (24%)	
2020–2025	641 (74%)	210 (82%)	431 (71%)	

^a^ Median (IQR); n (%).

^b^ Wilcoxon rank sum test; Pearson’s Chi-squared test; Fisher’s exact test.

Among the five PPI treatments, esomeprazole had the highest number of cases, accounting for 307 (36%), while rabeprazole had the fewest, with only 15 cases (1.7%). Additionally, our analysis indicated that age (*p* = 0.4) and gender (*p* = 0.5) may not be direct factors influencing fatality in both the fatal and non-fatal groups associated with PPI-related tumor events. The rates of fatalities did not significantly change between these groups across different PPI treatments (*p* = 0.2).

**FIGURE 4:**
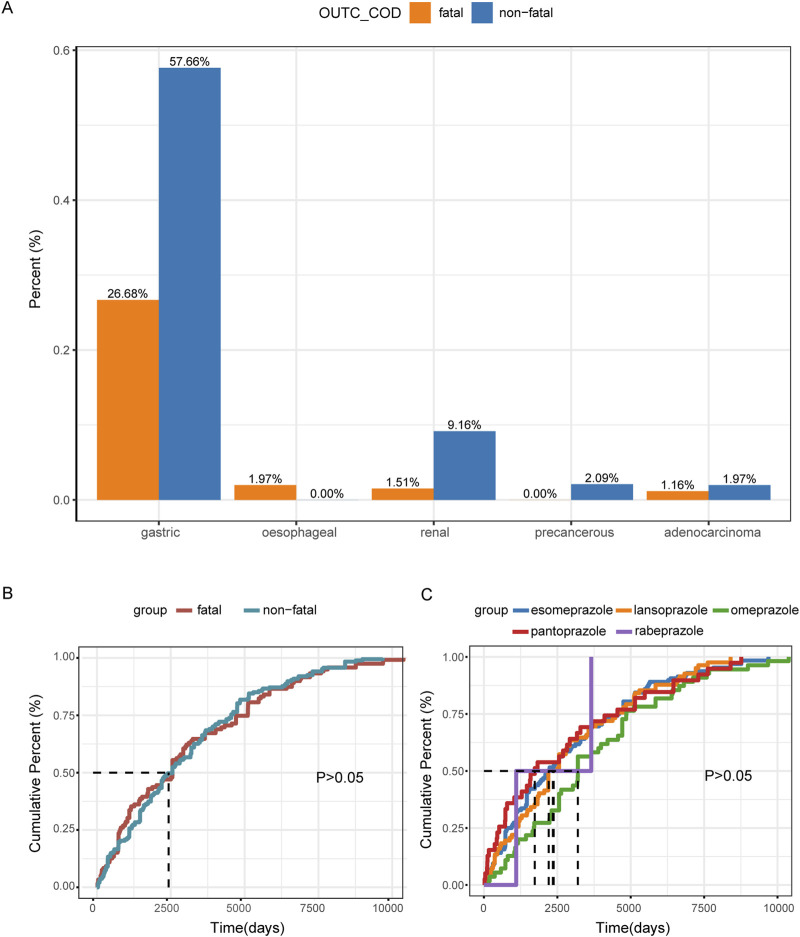
Descriptive analysis of reported TAEs associated with PPIs. **(A)** The bar chart presents the statistics of tumor occurrence sites reported in tAE cases. **(B)** The cumulative distribution curve shows the onset time of TAEs for the fatal group versus the non-fatal group. **(C)** The cumulative distribution curve displays the onset time of TAEs across different PPI treatment groups.

We also assessed the proportions of tumor sites related to PPIs and the associated fatality events. The highest proportion of tumor cases was gastric cancer, which included types such as gastric cancer, gastric adenocarcinoma, metastatic gastric cancer, and gastric cancer stage III. Notably, gastric cancer also accounted for the highest proportion of fatalities related to PPI use, representing 26.68% of fatality events (Figure 4A).

### Time to onset analysis

In our analysis, we observed no significant difference in the median time to onset of TAEs between the fatal and non-fatal groups. Specifically, the median time to onset was 2,556 days (IQR: 797–4,908) for the fatal group and 2,556 days (IQR: 1,096–4,547) for the non-fatal group, with *p* > 0.05 (Figure 4B; Table 1). Furthermore, the different PPI treatments did not appear to affect the median time to onset of TAEs, as indicated by *p* > 0.05 (Figure 4C).

### Factors influencing TAEs in PPIs

**FIGURE 5:**
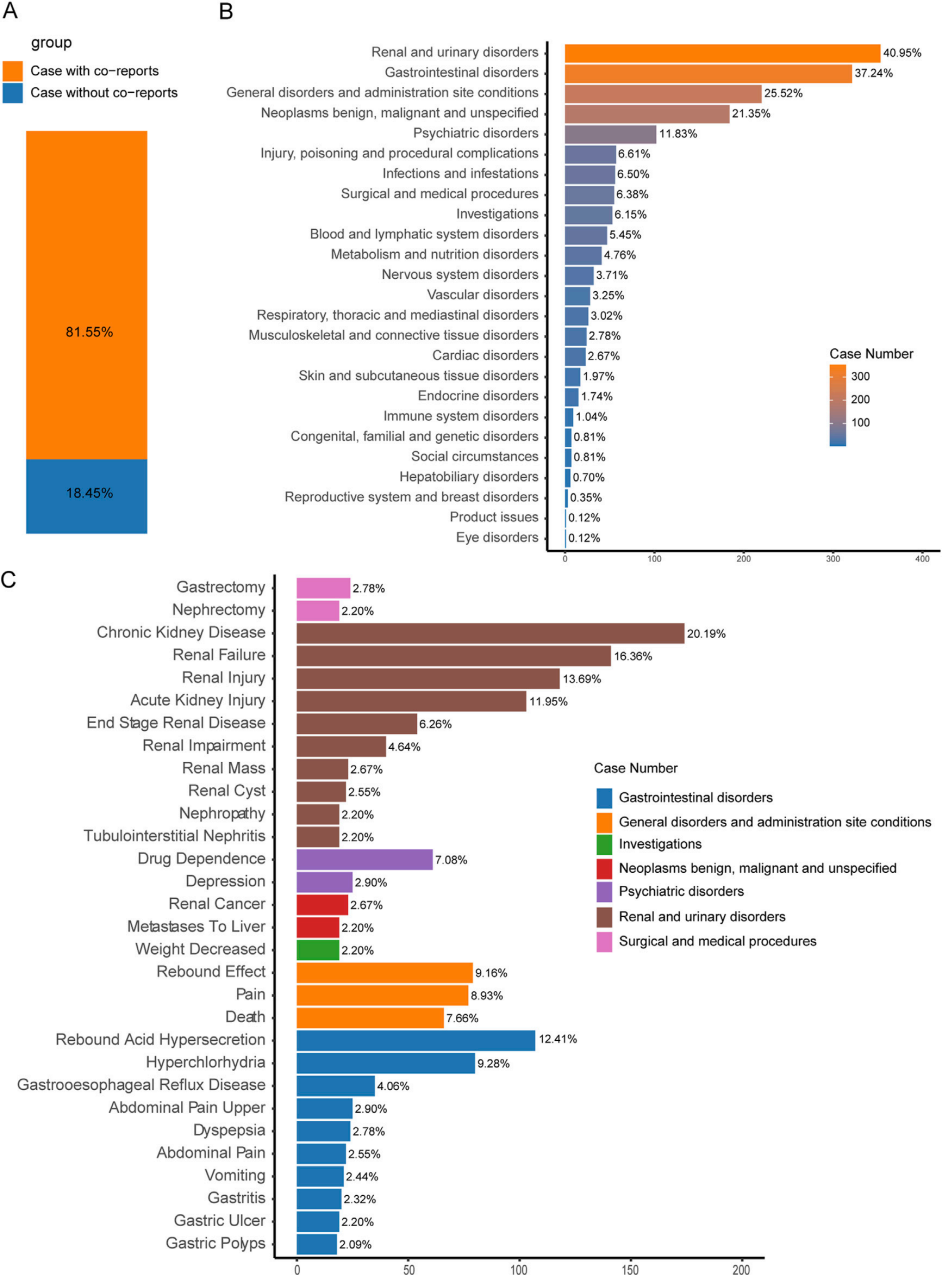
Factors influencing PPI-related TAEs. **(A)** The bar chart shows the proportion of cases with and without co-reported AEs among PPI-related tAE cases. **(B)** The bar chart displays the statistics of the SOC regarding PTs of co-reported AEs. The percentages indicated in the chart represent the proportion of cases with such AEs out of the total number of PPI-related tAE cases with concurrent adverse reactions. **(C)** The bar chart presents the statistics of the top 30% PTs of co-reported AEs. The colors indicate the corresponding SOC of each PT. The percentages indicated in the chart represent the proportion of cases with such AEs out of the total number of PPI-related tumor adverse event cases with concurrent adverse reactions.

We also investigated co-reported AEs that could potentially influence the occurrence of TAEs in PPIs. Among the 862 cases of PPI-associated TAEs we analyzed, 81.55% were accompanied by other AEs, while 18.45% involved only PPI-associated TAEs (Figure 5A). The three most commonly reported co-occurring AEs were renal and urinary disorders (40.95%), gastrointestinal disorders (37.24%), and general disorders and administration site conditions (25.52%) (Figure 5B).

Furthermore, within the reported cases of PPI-related TAEs, the development of certain conditions—such as renal mass (2.67%), renal cyst (2.55%), nephropathy (2.20%), tubulointerstitial nephritis (2.20%), gastroesophageal reflux disease (4.06%), and gastric ulcer (2.20%)—may be linked to the occurrence of tumors (Figure 5C).

Based on the reports of TAEs associated with PPIs, we carried out a univariate logistic regression analysis to acquire a deeper understanding of potential influencing factors. Compared to patients under 65 years old, those aged 65–75 years had a lower OR of experiencing PPI-related TAEs (OR = 0.91 [0.87, 0.95], *p* < 0.001). Similarly, patients over 75 years old exhibited lower odds compared to those under 65 years old (OR = 0.93 [0.89, 0.98], *p* = 0.004). Male patients had slightly higher odds of experiencing PPI-associated TAEs than female patients (OR = 1.06 [1.03, 1.09], *p* < 0.001). In addition, apart from the pantoprazole treatment group, other PPI treatments did not show a significant impact on the occurrence of PPI-related TAEs (Table 2). Using the FAERS database, we analyzed three dosage subgroups for five PPIs. In the Pantoprazole group, both the Medium Dose (21–40 mg/day) and High Dose (>40 mg/day) showed a significantly higher risk compared to the Low Dose (≤20 mg/day), with ORs of 1.16 (95% CI: 1.01–1.32, *p* < 0.05) for the Medium Dose and 1.19 (95% CI: 1.00–1.42, *p* < 0.05) for the High Dose (Table 3). However, for other drug groups, no significant association was found between dosage and the risk of TAEs.

**TABLE 2:** The factors influencing PPI-related TAEs. The considered exposure factors include gender, age, and different PPIs.

Characteristic	N	Exp (Beta)	95% CI^a^	P-value
**Age**	1,887			
<65 (Reference)		—	—	
65–75		0.91	0.87, 0.95	<0.001
>75		0.93	0.89, 0.98	0.004
**Sex**	2,641			
female (Reference)		—	—	
male		1.06	1.03, 1.09	<0.001
**Group**	3,140			
esomeprazole (Reference)		—	—	
lansoprazole		1.03	0.99, 1.07	0.141
omeprazole		0.97	0.93, 1.01	0.155
pantoprazole		0.93	0.89, 0.97	<0.001
rabeprazole		0.98	0.88, 1.09	0.728

^a^ CI, confidence interval.

^*^P < 0.05; ^**^P < 0.01; ^***^P < 0.001; ^****^P < 0.0001.

**TABLE 3:** Correlation between TAE occurrence and PPI dosage levels.

Characteristic	N	Exp (Beta)	95% CI^a^	P-value
**All**	838			
Low Dose (≤20 mg/day)	324	—	—	
Medium Dose (21–40 mg/day)	414	0.97	0.92, 1.03	0.394
High Dose (>40 mg/day)	100	1.00	0.91, 1.10	0.975
**Esomeprazole**	102			
Low Dose (≤20 mg/day)	28	—	—	
Medium Dose (21–40 mg/day)	70	0.96	0.79, 1.18	0.726
High Dose (>40 mg/day)	4	0.73	0.45, 1.17	0.188
**Lansoprazole**	329			
Low Dose (≤20 mg/day)	153	—	—	
Medium Dose (21–40 mg/day)	119	1.03	0.93, 1.15	0.550
High Dose (>40 mg/day)	57	1.05	0.92, 1.20	0.441
**Omeprazole**	139			
Low Dose (≤20 mg/day)	99	—	—	
Medium Dose (21–40 mg/day)	35	0.79	0.68, 1.12	0.132
High Dose (>40 mg/day)	5	0.77	0.54, 1.09	0.139
**Pantoprazole**	258			
Low Dose (≤20 mg/day)	34	—	—	
Medium Dose (21–40 mg/day)	190	1.16	1.01, 1.32	0.034
High Dose (>40 mg/day)	34	1.19	1.00, 1.42	0.047

^a^ CI, confidence interval.

## Discussion

PPIs, while effective and generally safe, have well-documented adverse reactions. However, comprehensive studies on TAEs linked to PPI use are lacking. Our study is the first pharmacovigilance research using FAERS database data to investigate TAEs associated with PPIs. We analyzed PPI-related AEs from FAERS spanning 2004 to the first quarter of 2024, specifically focusing on TAEs. By utilizing the complete FAERS database, we eliminated duplicate cases and excluded potential factors that could induce TAEs. We identified genuine TAEs using standardized MedDRA queries and disproportionality analysis. Our study found ten TAEs significantly associated with PPI treatment and examined their clinical characteristics. These findings are consistent with previous cohort studies, making this the largest real-world data study on PPI-related TAEs to date. We identified over 800 cases of TAEs linked to PPI use and analyzed incidence rates, demographic characteristics, tumor sites, and onset times for commonly used PPIs such as esomeprazole, lansoprazole, omeprazole, pantoprazole, and rabeprazole. Although tumor occurrences are a small fraction of PPI-related AEs, our study provides valuable insights into potential tumor risks with long-term PPI use.

In our study results, ten types of TAEs were significantly associated with PPI treatment. There were also differences among the five commonly used PPIs, with esomeprazole being associated with the highest number of cases and rabeprazole with the lowest. In the cases of PPI-related TAEs, the median age of onset for patients was 59 years, with a higher proportion of AEs occurring in males. However, in the comparison between the mortality group and the non-mortality group of cases with PPI-related TAEs, age and gender did not appear to be direct factors influencing mortality. Interestingly, there were no significant differences in the types of PPI treatments between the mortality and non-mortality groups. Our findings indicate that TAEs associated with PPIs are less likely to occur in elderly patients. This could be due to several factors. First, metabolic changes in older adults might alter the pharmacokinetics of PPIs, reducing the likelihood of adverse events. Additionally, older patients often take multiple medications, which can lead to drug interactions that either mitigate or mask the effects of PPIs. There may also be reporting biases, with adverse events in older patients being underreported. Further research is necessary to explore these hypotheses and to fully understand the mechanisms behind the age-related differences in the occurrence of TAEs.

Previous case reports have indicated that patients infected with *H. pylori* developed adenocarcinoma due to multiple hyperplastic polyps during long-term treatment with PPIs (Anjiki et al., 2017). This suggests that PPI-related tumor events are likely during PPI treatment. Supporting evidence for the association between PPI use and tumors includes biological mechanisms. Long-term PPI use can induce hypergastrinemia, leading to proliferative changes in the gastric mucosa, driven by gastrin’s central role in gastric acid regulation (Lundell et al., 2015; Blair et al., 1987). Gastrin has a trophic effect on enterochromaffin-like (ECL) cells. Jianu C S et al. proposed that varying degrees of hypergastrinemia, due to individual differences and variations in PPI treatment doses and durations, may progress to ECL cell neuroendocrine tumors through stages of ECL cell hyperplasia and atypical hyperplasia (Fossmark et al., 2019; Cavalcoli et al., 2015). Additionally, the profound acid-suppressive effects of PPIs may induce gastric neuroendocrine carcinoma from ECL cells (Jianu et al., 2012). Oxyntic atrophy can cause significant hypergastrinemia and the development of ECL cell neoplasia and adenocarcinoma. Similarly, long-term use of effective acid suppressants like PPIs can lead to varying degrees of malignant transformation of ECL cells (Waldum and Fossmark, 2021). PPI treatment in patients with *H. pylori* infection can result in more pronounced hypergastrinemia (Martinsen et al., 2003). Hypergastrinemia is a risk factor for the malignant progression of gastric hyperplastic polyps (Bordi et al., 1984), and adenocarcinoma can develop from these polyps, with an incidence of 2.1% observed in resected polyps (Yao et al., 2002). One case report described a patient with significant hypergastrinemia due to long-term PPI use and *H. pylori* infection, who developed adenocarcinoma from multiple hyperplastic polyps (Anjiki et al., 2017).

The biological mechanism by which long-term PPI use may lead to esophageal adenocarcinoma is related to its effect on gastrin levels. COX-2, a pro-inflammatory enzyme, promotes esophageal adenocarcinoma; normal gastrin levels may mitigate esophageal cancer risk by counteracting COX-2 activity, while elevated gastrin levels may upregulate COX-2 expression (Abdalla et al., 2004; Shirvani et al., 2000; Song et al., 2007). Animal models show that gastric juice can dose-dependently counteract bile acid reflux in rats, and esophageal exposure to bile acid reflux may lead to Barrett’s esophagus and subsequent adenocarcinoma (Nishijima et al., 2004; Ireland et al., 1996). Chronic kidney disease (CKD) is an established risk factor for renal cell carcinoma (Lowrance et al., 2014), and studies have shown a strong correlation between PPI use and the onset of CKD (Lazarus et al., 2016; Klatte et al., 2017).

Furthermore, it cannot be entirely ruled out that the progression of the disease itself may lead to tumors. For instance, some common gastric diseases can also progress to gastric cancer, with *H. pylori* recognized as one of the causes of gastric cancer, particularly gastric adenocarcinoma (Cancer, 1994; Plummer et al., 2015). In standardized treatment for *H. pylori* eradication, PPIs are often utilized. Epidemiological studies have reported that PPI treatment for gastroesophageal reflux following the eradication of *H. pylori* may induce gastric cancer (Cheung et al., 2018; Cheung and Leung, 2019). In summary, whether PPIs are related to these tumor adverse reactions and the mechanisms behind this relationship requires further research for exploration and verification.

In light of our findings, clinicians must carefully consider the type, dosage, and duration of PPI treatment to reduce the risk of adverse events. Treatment should be evaluated individually, ensuring alignment with clear, evidence-based guidelines. Our study shows a higher risk of adverse events with increased doses of pantoprazole, so clinicians should be especially cautious with these prescriptions. To address these risks, regular monitoring is crucial, particularly for patients on long-term PPI therapy. This involves periodic assessments of gastric health and attention to any new symptoms that could indicate side effects. Routine follow-ups and appropriate use of tools like endoscopy can aid in early detection and intervention, potentially lowering the risk of serious outcomes. By adopting these strategies, healthcare providers can balance the benefits of PPIs with their safety concerns, ultimately improving patient care.

This study also has certain limitations. First, the majority of the research data in the FAERS database, a worldwide spontaneous reporting system, originates from the United States. The COVID-19 pandemic led to the closure of some healthcare institutions worldwide and limited medical resources in 2020, resulting in delays in cancer diagnosis and treatment. Consequently, data related to cancer diagnoses and resulting mortality may be relatively outdated. Additionally, the database does not provide information on other risk factors for reported cases, such as diet, smoking, alcohol consumption, obesity, family history, and potential confounding factors, including other comorbidities of the reported cases.

In conclusion, this study conducted a comprehensive statistical analysis of AEs related to tumors that are highly correlated with PPIs, based on real data from the FAERS database. Although the proportion of PPI-related TAEs is relatively low among all reported PPI AEs, our study supports the existence of such events. Simultaneously, the results provide a foundation for understanding the potential TAEs associated with PPIs and help clinicians focus on the proper usage of PPIs and the complications they may provoke, especially with long-term usage. While PPIs are widely used for conditions such as gastric and duodenal ulcers, functional dyspepsia, prevention of ulcers during antiplatelet or steroid therapy, and stress-induced ulcers, their use should be assessed for limitations in cases without clear indications, necessitating further clarification on the indications for stress-induced ulcer treatment. Since this study involves only a statistical analysis of reported cases in the existing database, it is essential to support our findings through larger-scale prospective clinical studies or large randomized controlled trials. These studies will aid in identifying the true incidence of PPI-related TAEs and in uncovering potential risk factors and biological mechanisms, thereby enhancing risk management in clinical practice.

## Data Availability

The original contributions presented in the study are included in the article/Supplementary Material, further inquiries can be directed to the corresponding author.
